# Prognostic impact of pneumonitis after durvalumab therapy in patients with locally advanced non-small cell lung cancer

**DOI:** 10.1007/s10637-021-01191-6

**Published:** 2021-10-11

**Authors:** Ari Nishimura, Akira Ono, Kazushige Wakuda, Takanori Kawabata, Michitoshi Yabe, Taichi Miyawaki, Eriko Miyawaki, Hiroaki Kodama, Naoya Nishioka, Nobuaki Mamesaya, Haruki Kobayashi, Shota Omori, Hirotsugu Kenmotsu, Tateaki Naito, Haruyasu Murakami, Hideyuki Harada, Toshiaki Takahashi

**Affiliations:** 1 Division of Thoracic Oncology, Shizuoka Cancer Center, Shizuoka, Japan; 2 Clinical Research Center, Shizuoka Cancer Center, Shizuoka, Japan; 3 Division of Radiation Oncology, Shizuoka Cancer Center, Shizuoka, Japan

**Keywords:** Durvalumab, Chemoradiotherapy, Pneumonitis, Locally advanced non-small cell lung cancer

## Abstract

*Background. *Prognostic data on Japanese patients receiving durvalumab after chemoradiotherapy (CRT) for locally advanced non-small cell lung cancer (LA-NSCLC) are insufficient. Whether pneumonitis has prognostic implications in patients with LA-NSCLC who have received durvalumab also remains unclear. *Methods.* We retrospectively assessed the data of 82 consecutive patients who had received durvalumab after CRT at our institution between May 2018 and August 2020. A multi-state model was used to establish the associations between co-variables and progression-free survival (PFS). *Results.* The median observation period for all the censored cases was 14.5 months (5.7–28.9 months), the median PFS was 22.7 months, and the 12-month PFS rate was 62.3% (95% CI: 50.2%-72.3%). The median percentage of the lung volume receiving a radiation dose in excess of 20 Gray (V20) was 22% (4%-35%). Thirteen patients (16%) had Grade 1 pneumonitis before receiving durvalumab, and 62 patients developed pneumonitis after durvalumab (Grades 1, 2, and 3 in 25 [30%], 32 [39%], and 4 [5%], respectively). Twenty-four patients (29%) completed the 1-year durvalumab treatment period, 16 patients (20%) were continuing to receive treatment, and 42 (51%) had discontinued treatment. In a multi-state analysis, patients with pneumonitis before durvalumab therapy had a poorer PFS than those without pneumonitis (HR: 4.29, *p* = 0.002). The development of Grade 2 or higher pneumonitis after durvalumab was not a significant prognostic factor for PFS (HR: 0.71, *p* = 0.852). *Conclusion.* Grade 2 or higher pneumonitis after durvalumab was not a prognostic factor of PFS in LA-NSCLC patients received durvalumab.

## Introduction

The standard treatment for unresectable locally advanced non-small cell lung cancer (LA-NSCLC) is definitive chemoradiotherapy (CRT) with durvalumab. The PACIFIC trial showed that additional treatment with durvalumab therapy for patients with controlled disease after CRT can further improve prognosis [1].

In the PACIFIC trial, the incidence of any-grade pneumonitis was 33.9% in all patients and 73.6% in the Japanese subgroup [2]. Horinouchi et al. reported an incidence of radiation pneumonitis after CRT of 72.9% among Japanese patients with Stage III NSCLC in a retrospective cohort study that was performed before durvalumab approval [3]. And this incidence was similar to the incidence of pneumonitis in the Japanese subgroup of the PACIFIC study [3].

Haratani et al. reported that the development of immune-related adverse events (irAE) was associated with improved overall survival (OS) and progression-free survival (PFS) in patients with advanced or recurrent NSCLC [4]. On the other hand, Suresh et al. reported that in patients with advanced NSCLC, the development of immune checkpoint inhibitor (ICIs)-related pneumonitis was associated with a worse survival outcome in patients receiving immunotherapy [5]. However, it is difficult to determine whether pneumonitis occurring during durvalumab administrated after CRT for LA-NSCLC is radiation pneumonitis or irAE pneumonitis, and it is unclear whether this pneumonitis has an impact on prognosis.

In the present study, we hypothesized that in locally advanced NSCLC patients who received durvalumab therapy following CRT, the presence of Grade 2 or higher pneumonitis with any causes might predict a poor outcome. Therefore, we evaluated the association between the presence of Grade 2 or higher pneumonitis and patient outcome using multi-state models to analyze time-to-event data.

## Patients and methods

### Patients

**Fig. 1:**
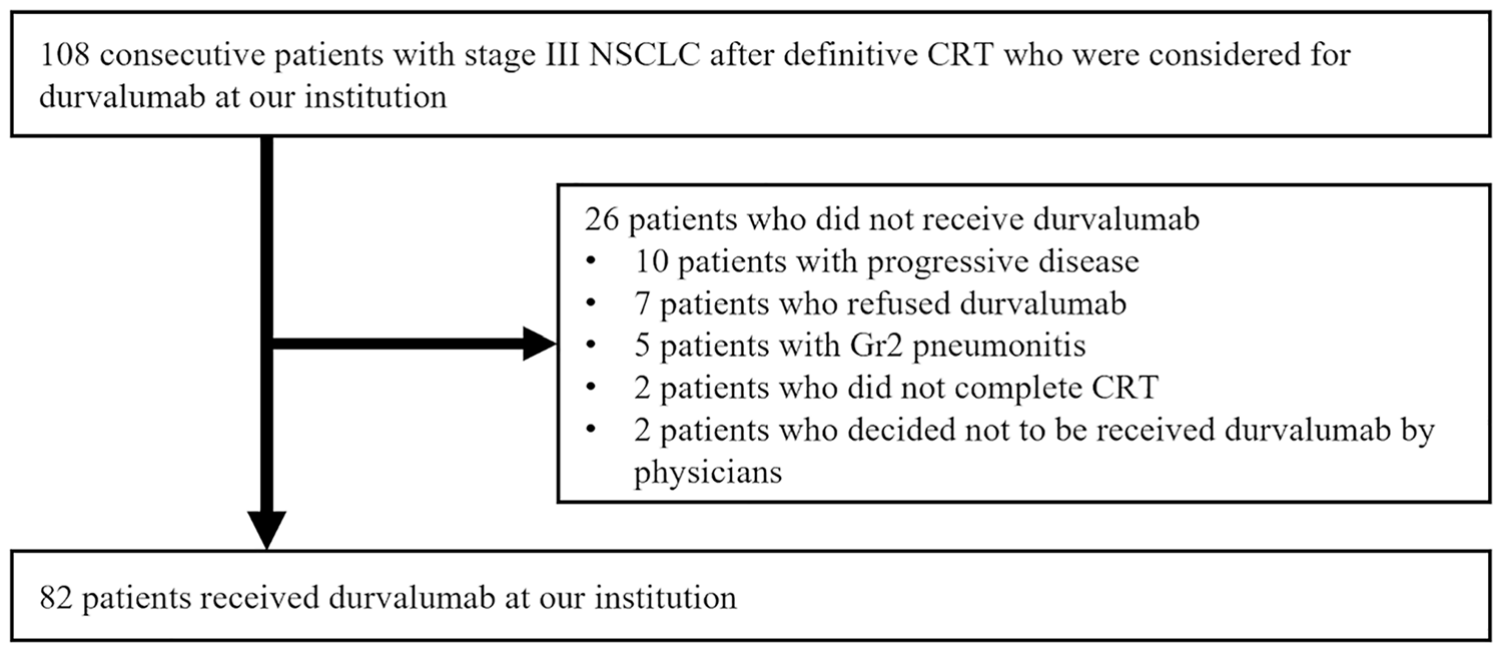
Flow diagram of the patients included in this study (*n* = 82). CRT: chemoradiotherapy; Dur: durvalumab; Gr: Grade

One-hundred and eight consecutive patients with stage III NSCLC after definitive CRT who were considered for durvalumab at our institution between April 2018 and January 2020, including 5 patients who were referred to our institution after CRT at other hospitals for durvalumab therapy. Of these 108 cases, 82 patients with no progression after CRT were started on durvalumab at our institution between July 2018 and March 2020 (Fig. 1). We retrospectively reviewed the data for these 82 patients. The indication for durvalumab therapy following CRT was determined by reviewing the patient condition including good ECOG PS, the absence of severe radiation pneumonitis of Grade 2 or higher in a multidisciplinary conference. Durvalumab was administered at a dosage of 10 mg/kg every 2 weeks until disease progression or intolerance, for up to 12 months. Fifty-five patients were treated with three-dimensional conformal radiation, and 14 patients were treated with intensity modulated radiation therapy (IMRT). Thirteen patients were treated with proton beam therapy (PBT) after approval was obtained at a multidisciplinary conference. We selected IMRT/PBT when the irradiation doses to normal tissue, such as lung and spinal cord tissues, were likely to exceed the acceptable range. The total radiation dose in all the patients was 60 Gray or 64 Gray.

We diagnosed pneumonitis based on CT or chest X-ray findings in patients with a fever, cough, or dyspnea or, in the absence of symptoms, incidentally during a follow-up examination. Patients with apparent pulmonary infection or heart failure were excluded. The grade of pneumonitis was evaluated according to the Common Terminology Criteria for Adverse Events (CTCAE), v.5.0. Patients with Grade 1 pneumonitis at baseline who did not experience an exacerbation after durvalumab administration were classified as Grade 0, and those with an exacerbation were classified as Grade 2 or higher. These criteria were consistent with those used in the Pacific trial.

Candidate variables for associations with Grade 2 or higher pneumonitis included sex, age (over 70 years), V20, pathological type, smoking history, presence of Grade 1 pneumonitis at baseline, type of radiation therapy (X-ray or proton beam), and location of the lesion (upper lobe or lower lobe).

### Statistical method

**Fig. 2:**
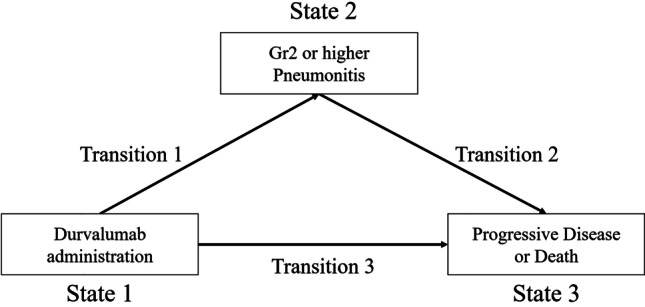
Diagram showing the multistate model used for modeling the impact of Grade 2 or higher pneumonitis on PFS after durvalumab administration following CRT in patients with NSCLC. Transition 1 is evaluating relationship between variables and occurring grade 2 or higher pneumonitis. Transition 2 is evaluating factor’s impact on PFS in patients with grade 2 pneumonitis. Transition 3 is evaluating factor’s impact on PFS in all patients. Gr: grade

We investigated transitions, including the time of durvalumab therapy, Grade 2 pneumonitis, disease progression, or death, using a multivariate “multi-state” model. Specifically, we used three states of illness and death without recovery, which is probably the simplest framework in the multi-state model. This model enabled us to evaluate the effects of the covariates for each transition taking into account the clinical course after Grade 2 pneumonitis. Each patient was classified into one of three states in a multi-state model: (1) transition from starting durvalumab therapy to the onset of Grade 2 pneumonitis, (2) transition from the onset of Grade 2 pneumonitis to disease progression or death including patients who died of other causes, and (3) transition from the start of durvalumab therapy until disease progression or death resulting from any cause (Fig. 2). For pneumonitis, we assessed the association between the onset of Grade 2 or higher pneumonitis and prognosis, taking into account the time from the administration of durvalumab to the onset of pneumonitis and each baseline factor. Disease progression was defined according to the RECIST criteria.

PFS was defined as the time from the day of the start of durvalumab therapy until disease progression or death resulting from any cause, whichever occurred first. V20 was treated as a continuous variable. Prognostic factors among baseline covariates were identified using the univariate and multivariate multi-state model. The prognostic significances of all the variables were measured by calculating the adjusted hazard ratio (HR) with a 95% confidence interval (95% CI). *P* values < 0.05 were considered to be indicative of statistical significance. All the statistical analyses were performed using R package, version 3.6.1, for Mac.

## Results

A flow-diagram of the patients included in the analysis is shown in Fig. 2. Of the 108 patients who received CRT for locally advanced NSCLC, 82 patients were included in this study and the remaining 26 patients were excluded: 2 patients did not complete CRT, 10 patients had progressive disease after CRT, 7 patients refused durvalumab therapy, 5 patients had Grade 2 radiation pneumonitis after CRT, and 2 patients were not considered to be candidates for treatment by their physicians. The characteristics of the 82 patients are summarized in Table 1. The median age was 69.5 years (range: 37–86 years). The subjects included ten patients who had received CRT for a mediastinal lymph node (#3a, #4 L, #4R, #7) recurrence; all ten of these patients were in the upper lobe lesion group. The concurrent chemotherapy regimen was cisplatin + S-1 in 24 patients, carboplatin + paclitaxel in 20 patients, cisplatin + vinorelbine in 18 patients, daily carboplatin in 11 patients, and carboplatin + nab-paclitaxel in 3 patients. The PD-L1 tumor proportion score (TPS) with the 22C3 assay was calculated in 46 patients; 17 of them had a PD-L1 TPS < 1%, and 11 of them had a PD-L1 TPS ≥ 50%. The median observation period for all the censored cases was 14.5 months (range: 5.7–28.9 months), and the 18-month PFS rate was 55.6% (95% CI: 42.5%-66.8%). Seventy patients (85.4%) were treated with durvalumab within 42 days of completion of CRT.

**Table.1:** Characteristics of all patients and patients with Gr 0/1 and Gr 2 or higher pneumonitis

		All	Gr 0/1	Gr 2-
	Number	82	43	39
Age (years)	≧70	17	12	5
	< 70	65	31	34
Sex	Male	54	41	13
	Female	28	2	26
Pathological type	Adeno	48	25	23
	Squamous	26	14	12
	Unknown	8	4	4
Clinical Stage	IIIA	30	17	13
	IIIB	29	12	17
	IIIC	13	6	7
	LN rec	10	8	2
Location	Upper lobe	59	43	16
	Lower lobe	23	9	14
Smoking history	Yes	67	34	33
	No	15	9	6
Baseline pneumonitis	Yes	13	8	5
	No	69	35	34
Radiation	X-ray	55	30	25
	IMRT	14	8	6
	Proton beam	13	5	8
V20 (continuous)	Median (range)	22 (5–34)	18 (5–34)	26 (10–35)

*Adeno* adenocarcinoma, *Squamous* squamous cell carcinoma, *LN rec* Lymph Node recurrence, *IMRT* intensity modulated radiation therapy, *V20* the percentage of lung volume receiving radiation dose in excess of 20 Gy

Thirteen of the 82 patients had Grade 1 pneumonitis at baseline, and the pneumonitis worsened to Grade 2 in 4 patients and to Grade 3 in 1 patient, and they all could not be re-administrated durvalumab therapy. Of the 69 patients with no pneumonitis at baseline, 56 patients developed pneumonitis after durvalumab administration: 25 patients had Grade 1, 28 patients had Grade 2, and 3 patients had Grade 3 pneumonitis, and of 56 patients, 30 patients (53.6%) could be re-administrated durvalumab therapy and 26 patients (46.4%) could not. Thus, pneumonitis developed after administration of durvalumab therapy in 61 patients (74.3%): Grade 1 in 25 (30.5%), Grade 2 in 32 (39.0%), and Grade 3 in 4 (4.9%) patients, respectively. In patients with baseline pneumonitis, the median interval from the completion of CRT to the administration of durvalumab therapy was 37 days (range: 15–99 days), and in patients without baseline pneumonitis, the median interval was 21 days (range: 5–72 days). The median percentage of V20 was 22% (range: 4%-35%).

The pneumonitis developed at a median interval of 2.1 months (range: 0.5–6.0 months) after the completion of radiotherapy and a median of 0.5 months (range: 0.0–2.6 months) after the final dose of durvalumab. Pneumonitis developed beyond the radiation field in 2 patients.

**Fig. 3:**
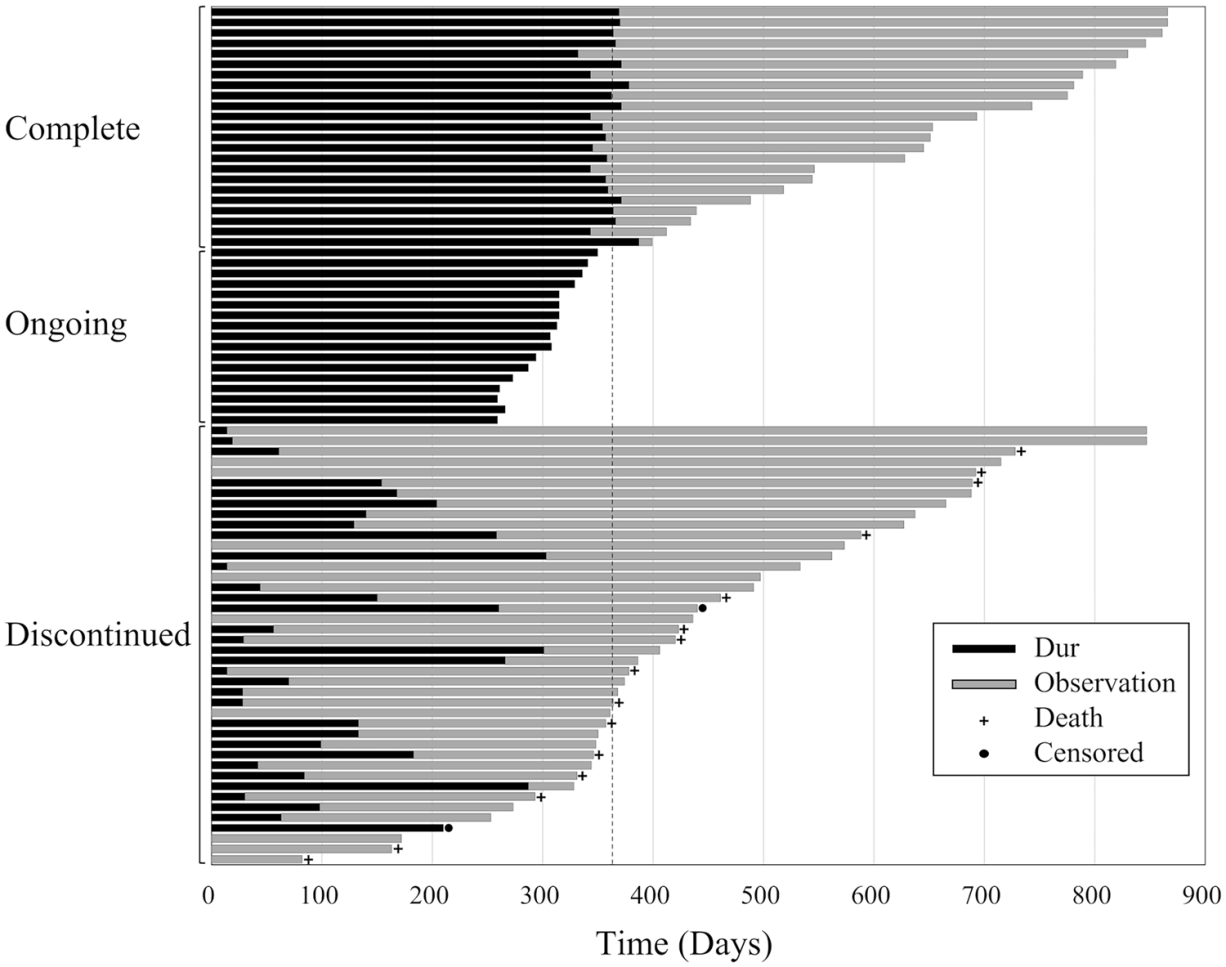
Durvalumab administration period and overall survival. Dur: durvalumab

Twenty-four patients (29.3%) completed the 1-year treatment period of durvalumab, 16 patients (19.5%) were receiving ongoing treatment, and 42 (51.2%) had discontinued treatment (Fig. 3). Of the 42 patients who had discontinued durvalumab therapy, 21 patients (50.0%) had pneumonitis, 18 patients (42.9%) had progressive disease, 2 patients (4.2%) had an exacerbation of chronic obstructive pulmonary disease, and 1 patient (2.1%) had a synchronous malignancy. Of the 42 patients who had discontinued durvalumab therapy, 15 patients died; in contrast, all the patients who completed the 1-year durvalumab treatment period were alive. An additional observation period was added to accurately determine the completion rate of durvalumab treatment, 38 patients (46.3%) completed the 1-year treatment period of durvalumab, and 44 patients (53.7%) had discontinued treatment.

Of the 36 patients with Grade 2–3 pneumonitis, 20 patients (55.6%) required prednisolone (PSL) therapy because their pneumonitis did not improve after durvalumab suspension, and 5 patients recovered rapidly in response to PSL and were able to continue receiving durvalumab. Of the 16 patients with Grade 2 pneumonitis, the PSL starting dose was 0.5 mg/kg in 11 patients and 1.0 mg/kg in 5 patients. Of the 4 patients with Grade 3 pneumonitis, 3 patients received methylprednisolone before PLS at a starting dose of 0.5 mg/kg and 1 patient received PSL at a starting dose of 1.0 mg/kg. Of the 6 patients who returned to durvalumab therapy after receiving PSL, 1 patient experienced an exacerbation of pneumonitis and durvalumab was re-suspended. The patient was given PSL again at a dose of 0.5 mg/kg, and the pneumonitis improved. A second re-administration of durvalumab was not performed. In 20 patients (Grade 1, 8; Grade 2, 12), durvalumab was re-administered after the pneumonitis was resolved. Of these 20 patients, 8 patients with an exacerbation of pneumonitis, durvalumab administration was discontinued.

First, each variable was tested in a univariate analysis. V20 (HR, 1.06; 95% CI, 1.02–1.11; *p* = 0.006) was significantly associated with Grade 2 or higher pneumonitis. Pneumonitis before durvalumab treatment (HR, 5.12; 95% CI, 1.52–17.2; *p* = 0.021) was a significant prognostic risk factor affecting the PFS. In the multivariate analysis, V20 (HR, 1.07; 95% CI, 1.02–1.13; *p* = 0.010) was significantly associated with Grade 2 or higher pneumonitis, and pneumonitis before durvalumab treatment (HR, 4.29; 95% CI, 1.23–14.88, *p* = 0.022) was a significant prognostic risk factor for PFS. The development of Grade 2 or higher pneumonitis after durvalumab was not a significant prognostic factor for PFS (HR: 0.71, p = 0.852). The development of Grade 2 or higher pneumonitis after durvalumab was not a significant prognostic factor for PFS (HR: 0.71, *p* = 0.852) (Table 2).

**Table.2:** Multi-state model results. Prognostic factors with respect to parameter estimates related each transition in multivariate analysis

	Transition (1)		Transition (2)		Transition (3)	
No. at risk	82		39		82	
No. of events	39		15		14	
	HR (95% CI)	*P*-value	HR (95% CI)	*P*-value	HR (95% CI)	*P-*value
Age						
< 70	1		1		1	
≧70	0.61 (0.23–1.63)	0.325	1.29 (0.39–4.27)	0.672	0.64 (0.07–5.58)	0.685
Sex						
Male	1		1		1	
Female	0.70 (0.30–1.61)	0.397	0.52 (0.16–1.67)	0.273	1.65 (0.36–7.52)	0.517
V20 (per unit increase)						
	1.07 (1.02–1.13)	0.010	1.04 (0.97–1.12)	0.264	0.99 (0.89–1.10)	0.809
Pathological type						
Sq	1		1		1	
Non-Sq	1.31 (0.59–2.93)	0.507	0.89 (0.26–3.10)	0.860	0.53 (0.14–1.96)	0.339
Smoking history						
Never	1		1		1	
Former/current	1.12 (0.35–3.58)	0.855	0.44 (0.12–1.66)	0.227	1.48 (0.17–12.86)	0.724
Tumor Location						
Upper	1		1		1	
Lower	0.89 (0.40–1.99)	0.780	0.63 (0.18–2.23)	0.475	1.93 (0.55–6.76)	0.303
Pneumonitis before Dur						
No	1		1		1	
Yes	1.02 (0.36–2.89)	0.977	0.48 (0.12–1.92)	0.301	4.29 (1.23–14.88)	0.022
Gr 2 pneumonitis						
No	1					
Yes	0.71 (0.02–26.65)	0.852		*P*-value = 0.852		

*HR* hazard ration, *CI* confidence interval, *V20* the percentage of lung volume receiving radiation dose in excess of 20 Gy, *Sq* squamous cell carcinoma, *Dur* durvalumab, *Gr* gray

## Discussion

We demonstrated that Grade 2 pneumonitis after durvalumab administration following CRT was not associated with a poor prognosis, while pneumonitis before durvalumab administration following CRT was associated with a poor PFS. In our study, the PFS was compatible with that observed in the Japanese subgroup of the PACIFIC trial [3], confirming the reliability of our study.

In the previous study, Inoue et al. suggested that the severity of pneumonitis after durvalumab administration was not associated with V20 or any other factors [6]. However, their small-scale study was a hypothesis-generating study, and we conducted a more reliable study by adding a number of cases and events and extending the observation period; as a result, we obtained different results regarding the relationship between V20 and pneumonitis. Furthermore, Tsujino et al. reported that V20 is associated with the incidence and grade of radiation pneumonitis in cases of lung cancer treated with concurrent chemotherapy [7], consistent with our results.

The incidence of pneumonitis in this study was similar to that in the Japanese subgroup of the PACIFIC study [2] as well as that in a retrospective Japanese study of chemoradiotherapy for unresectable Stage III NSCLC [3]. Horinouchi et al. reported that CRT-related pneumonitis occurred about 10–12 weeks after the completion of CRT in patients with NSCLC [3], consistent with the time of onset of pneumonitis in the present study. Pneumonitis induced with ICI, including durvalumab, occurred at a rate of 1.0% in a phase I/II trial [8]. Saito et al. reported that the incidence of Grade 2 or higher pneumonitis after CRT followed by durvalumab did not significantly differ from that of patients treated with CRT alone [9]. On the other hand, Shaverdian et al. reported that patients treated with CRT and durvalumab developed severer radiation pneumonitis than patients treated with CRT alone [10]. CT findings characteristic of irAE pneumonitis, such as the reversed halo sign, have been reported in pneumonitis observed during ICI therapy [11]. In this study, 1 case who diagnosed irAE pneumonitis with reversed halo sign was included. The patient had pneumonitis with reversed halo sign in the radiation field, which was diagnosed irAE pneumonitis and responded rapidly to steroid therapy. In most cases, it was difficult to distinguish between radiation pneumonitis and irAE pneumonitis. Although the interval time between completion of CRT and administration of durvalumab therapy and whether the pneumonitis shadow is in the irradiation field are important points for differentiation, in case with findings such as the reversed halo sign, which is characteristic of irAE pneumonitis, the possibility of irAE pneumonitis should be considered even if it is in the irradiation field.

Considering the above report of radiation pneumonitis after CRT, it is possible that the majority of pneumonitis cases occurring after the administration of durvalumab were radiation pneumonitis caused by CRT. Asians have a higher incidence of radiation pneumonitis compared to other races [12]. Lee et al. reported that pharmacokinetics of ICI in Asian were comparable to other races [13]. Radiation pneumonitis might be a strong component of pneumonitis after the administration of durvalumab, and although a previous study reported that severe radiation pneumonitis was associated with a poor prognosis among patients with advanced-stage lung cancer [14], the current study did not find an association between Grade 2 or higher pneumonitis and PFS. However, previous reports of the prognostic impact of pneumonitis have not taken into account changes in hazards for progression caused by pneumonitis [15], and the results should be interpreted with caution. Therefore, our study might be appropriate, since our results took time-to-event data into account using a multi-state analysis.

Initially, patients with radiation pneumonitis at the time of durvalumab administration were not included in the PACIFIC study; during the course of the trial, however, the protocol was revised to include pneumonitis up to Grade 1 [1]. However, the safety of ICIs in patients with Grade 1 radiation pneumonitis has not been confirmed. Considering that complications associated with Grade 1 baseline radiation pneumonitis have an impact on a shortened PFS, patient selection for durvalumab therapy following CRT for patients with advanced NSCLC might need to be performed with caution. Furthermore, since durvalumab treatment was discontinued within 3 months in several cases, the causes of treatment discontinuation should be addressed in future studies.

This study had some limitations. First, this was a retrospective study performed at a single institution with a relatively small number of patients. Therefore, the 95% CI of the HR for each transition was relatively wide because of the small number of events. Second, the follow-up period was too short to draw any conclusions regarding prognosis. However, our study provides important information on the mid-term patient prognosis and safety of durvalumab treatment. Although all the cases of pneumonitis that developed in this study occurred within 3 months after the final durvalumab dose, pneumonitis is likely to develop at some time during the follow-up period. Further study with a longer follow-up period is needed to confirm our results.

## Conclusion

Grade 2 or higher pneumonitis after durvalumab administration following CRT was not associated with PFS in LA-NSCLC. However, Grade 1 radiation pneumonitis before durvalumab administration was an unfavorable prognostic factor.

## References

[1] Antonia SJ, Villegas A, Daniel D (2017). Durvalumab after Chemoradiotherapy in Stage III Non-Small-Cell Lung Cancer. N Engl J Med.

[2] Faehling M, Schulz C, Laack H et al (2019) PACIFIC subgroup analysis: pneumonitis in stage III, unresectable NSCLC patients treated with durvalumab vs. placebo after CRT. München, p s-0039–1678247

[3] Horinouchi H, Atagi S, Oizumi S (2020). Real-world outcomes of chemoradiotherapy for unresectable Stage III non-small cell lung cancer: The SOLUTION study. Cancer Med.

[4] Haratani K, Hayashi H, Chiba Y (2018). Association of Immune-Related Adverse Events With Nivolumab Efficacy in Non–Small-Cell Lung Cancer. JAMA Oncol.

[5] Suresh K, Psoter KJ, Voong KR (2019). Impact of Checkpoint Inhibitor Pneumonitis on Survival in NSCLC Patients Receiving Immune Checkpoint Immunotherapy. J Thorac Oncol.

[6] Inoue H, Ono A, Kawabata T (2020). Clinical and radiation dose-volume factors related to pneumonitis after treatment with radiation and durvalumab in locally advanced non-small cell lung cancer. Invest New Drugs.

[7] Tsujino K, Hirota S, Endo M et al (2003) Predictive value of dose-volume histogram parameters for predicting radiation pneumonitis after concurrent chemoradiation for lung cancer. Intern J Rad Oncol Biol Phys 55:110–115. 10.1016/S0360-3016(02)03807-5 10.1016/s0360-3016(02)03807-5 12504042

[8] Rizvi NA, Brahmer JR, Ou S-HI (2015). Safety and clinical activity of MEDI4736, an anti-programmed cell death-ligand 1 (PD-L1) antibody, in patients with non-small cell lung cancer (NSCLC). JCO.

[9] Saito S, Abe T, Kobayashi N (2020). Incidence and dose-volume relationship of radiation pneumonitis after concurrent chemoradiotherapy followed by durvalumab for locally advanced non-small cell lung cancer. Clinical and Translational Radiation Oncology.

[10] Shaverdian N, Thor M, Shepherd AF (2020). Radiation pneumonitis in lung cancer patients treated with chemoradiation plus durvalumab. Cancer Med.

[11] Nakashima K, Naito T, Omori S (2016). Organizing Pneumonia Induced by Nivolumab in a Patient with Metastatic Melanoma. J Thorac Oncol.

[12] Niu X, Li H, Chen Z (2012). A study of ethnic differences in TGFβ1 gene polymorphisms and effects on the risk of radiation pneumonitis in non-small-cell lung cancer. J Thorac Oncol.

[13] Lee K-W, Lee DH, Kang JH (2018). Phase I Pharmacokinetic Study of Nivolumab in Korean Patients with Advanced Solid Tumors. Oncologist.

[14] Inoue A, Kunitoh H, Sekine I et al (2001) Radiation pneumonitis in lung cancer patients: a retrospective study of risk factors and the long-term prognosis. Intern J Rad Oncol Biol Phys 49:649–655. 10.1016/S0360-3016(00)00783-5 10.1016/s0360-3016(00)00783-5 11172945

[15] Desilets A, Blanc-Durand F, Lau S (2021). Durvalumab therapy following chemoradiation compared with a historical cohort treated with chemoradiation alone in patients with stage III non–small cell lung cancer: A real-world multicentre study. Eur J Cancer.

